# Reduction of Wrong-Limb Blood Pressure Cuff Placement Attempts With a High-Visibility Limb Alert Sleeve: A Randomized Crossover Simulation Study

**DOI:** 10.7759/cureus.110913

**Published:** 2026-06-15

**Authors:** Taylor Florio, Gina Santosuosso, Anthony Dardano

**Affiliations:** 1 Plastic and Reconstructive Surgery, Larkin Community Hospital Palm Springs Campus, Hialeah, USA; 2 Plastic Surgery Nursing, Delray Medical Center, Delray, USA; 3 Plastic and Reconstructive Surgery, Delray Medical Center, Delray, USA

**Keywords:** medical device safety, medical device trials, preventable injury, quality improvement and patient safety, wrong-site surgery

## Abstract

Background: Wrong-limb blood pressure (BP) measurement is a persistent and preventable clinical error, particularly in patients with limb restrictions such as arteriovenous fistulas, post-mastectomy status, or vascular compromise. Despite documentation in electronic medical records, adherence to limb alerts remains inconsistent, placing patients at risk for complications including fistula thrombosis, lymphedema exacerbation, and vascular injury. Reliance on chart review alone may be insufficient in fast-paced clinical environments. This study evaluated whether a high-visibility limb alert sleeve could improve adherence to limb restrictions and reduce wrong-limb BP cuff placement attempts.

Methods: We conducted a randomized, within-subject crossover simulation study involving 50 clinical staff members, including nurses and ancillary personnel, recruited from inpatient, intensive care unit, surgical, and emergency department settings. Each participant completed both the chart-only and sleeve-present study conditions and therefore served as their own control. Participants obtained vital signs during two simulated patient encounters under different study conditions: (1) chart-only, in which limb restrictions were documented solely within the patient chart, and (2) sleeve-present, in which a high-visibility limb alert sleeve was placed on the restricted arm in addition to chart documentation. The order of conditions was randomized to minimize learning effects. The primary outcome was the rate of wrong-limb BP cuff placement attempts. Secondary outcomes included spontaneous recognition and correction of incorrect initial attempts. Paired statistical analysis was performed using McNemar’s test, with significance defined as p < 0.05.

Results: Wrong-limb BP cuff placement attempts occurred in 30 of 50 encounters (60%) in the chart-only condition compared with one of 50 encounters (2%) in the sleeve-present condition (p < 0.0001). The paired absolute reduction in wrong-limb BP cuff placement attempts associated with the sleeve-present condition was 58.0% (95% CI: 44.3%-71.7%). The single incorrect attempt observed in the sleeve-present condition was recognized and corrected prior to cuff inflation.

Conclusions: In this simulated clinical study, high-visibility limb alert sleeves significantly reduced wrong-limb BP cuff placement attempts compared with chart-based documentation alone. By providing an immediate visual cue at the point of care, the intervention may help address an important gap in recognition of limb restrictions during routine clinical workflow. Given its straightforward design and visibility at the point of care, the limb alert sleeve may represent a practical strategy to reduce preventable wrong-limb BP cuff placement attempts in patients with limb restrictions. Further studies in real-world clinical settings are warranted to confirm these findings and evaluate long-term effectiveness across broader patient populations.

## Introduction

Wrong-limb blood pressure (BP) measurement represents a preventable clinical safety error in patients with limb restrictions despite widespread use of electronic medical records (EMRs) and other documentation systems [1,2]. Patients with limb restrictions, including those with arteriovenous fistulas, prior axillary surgery, lymphedema risk, or vascular compromise, are commonly identified as requiring avoidance of BP measurements on the affected limb because of concerns regarding potential complications. Potential complications may include fistula thrombosis, worsening lymphedema, pain, and vascular injury [3,4]. Although the strength of evidence supporting these practices may vary among clinical populations, limb restrictions remain widely used in routine patient care.

Although limb restrictions are commonly documented in EMRs, reliance on chart review alone may be insufficient in fast-paced clinical environments where clinicians often prioritize immediate clinical tasks [5]. Cognitive load, workflow interruptions, and alert fatigue may further contribute to missed safety cues and procedural errors [5,6].

Visual reminder systems have improved adherence to safety protocols in other healthcare settings [7-9], but the use of highly visible physical reminder devices to reduce wrong-limb BP measurement attempts in patients with limb restrictions has not been well studied. In this study, we evaluated whether a high-visibility limb alert sleeve reduces wrong-limb BP cuff placement attempts, the primary study outcome, compared with chart-based documentation alone in a simulated clinical environment.

## Materials and methods

We conducted a randomized within-subject crossover simulation study involving 50 clinical staff members recruited from inpatient, intensive care unit (ICU), surgical, and emergency department clinical settings at Larkin Community Hospital. This single-center study was conducted during March 2026. Eligible participants included nurses and ancillary clinical personnel with prior experience obtaining routine vital signs in patient care environments. Participation was voluntary and uncompensated. Each participant completed both the chart-only and sleeve-present study conditions and therefore served as their own control. The study was reviewed by the Larkin Community Hospital Institutional Review Board and was determined to be exempt from formal review (IRB #R-0426QI/TF).

Each participant completed two simulated BP measurement scenarios under different study conditions. In the chart-only condition, limb restrictions were documented solely within the simulated patient chart. In the sleeve-present condition, a high-visibility limb alert sleeve was placed on the restricted arm in addition to standard chart-based documentation.

**Figure 1:**
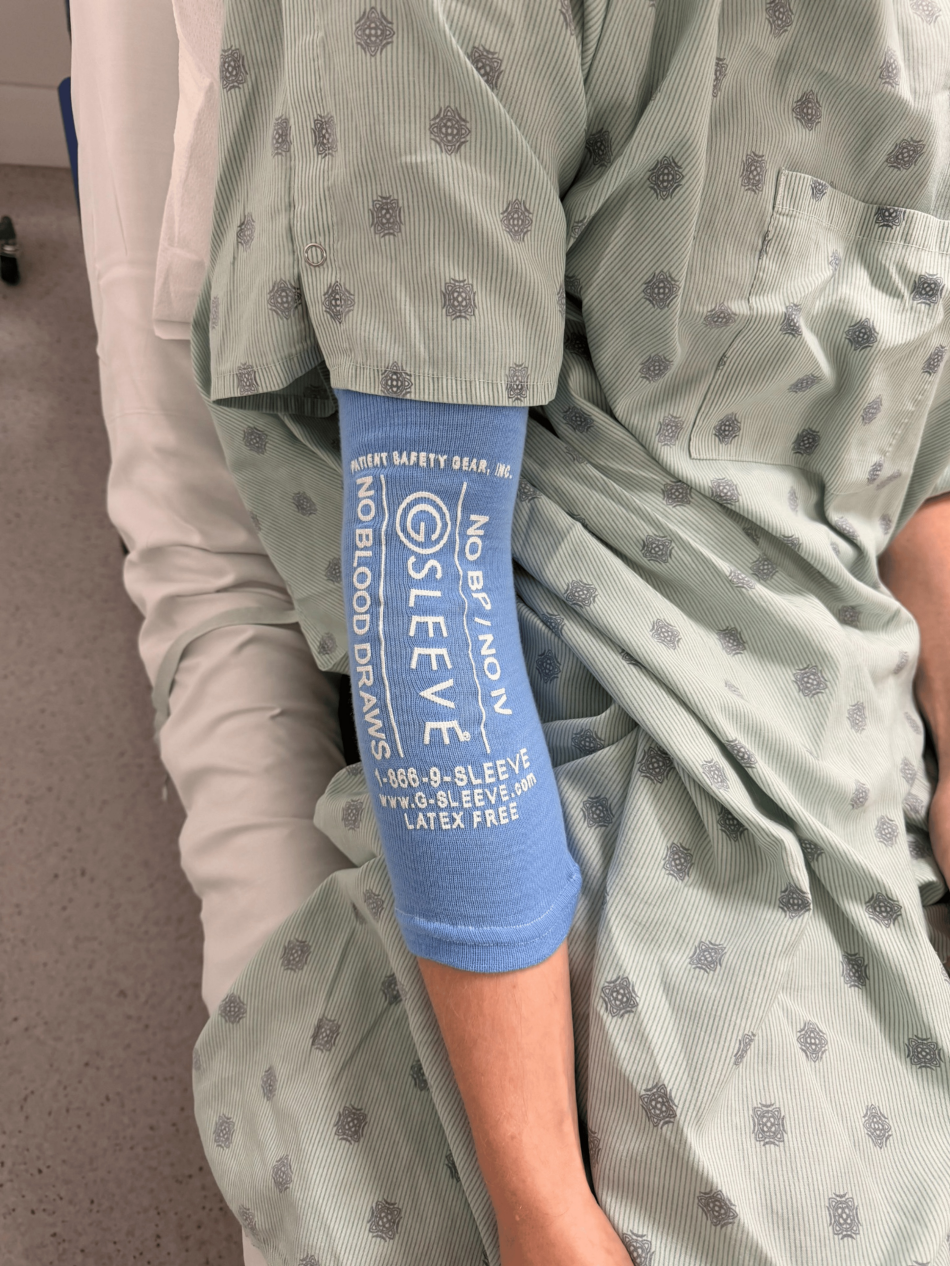
High-Visibility Limb Alert Sleeve Representative high-visibility limb alert sleeve placed on the restricted arm during the sleeve-present study condition.

The limb alert sleeve used in this study was designed to function as a passive visual safety cue and to remain highly visible at the point of care during routine clinical workflow. The sleeve was positioned on the restricted upper extremity to maximize visibility during routine BP assessment. A representative image of the limb alert sleeve is shown in Figure 1.

The order of study conditions was randomized at the time of participant enrollment. Participants served as their own controls. To minimize carryover effects, participants were not informed of the study hypothesis before completion of both scenarios, and no corrective feedback was provided between conditions.

**Figure 2:**
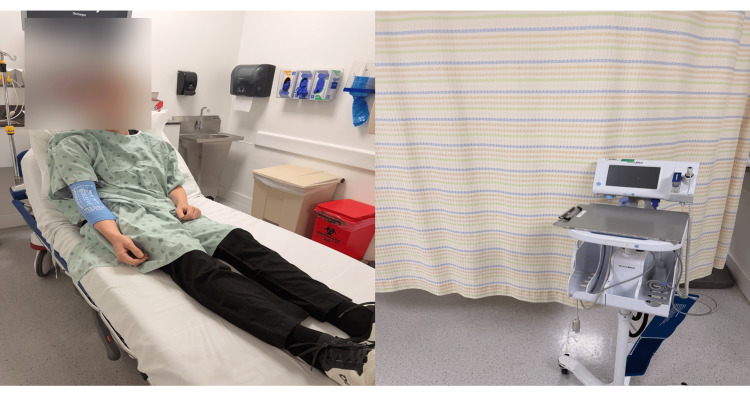
Simulation Study Setup Representative simulation environment demonstrating the simulated patient encounter and placement of the mock patient chart on the blood pressure machine outside the patient room prior to vital sign assessment.

Participants were provided a printed mock patient chart immediately outside the simulated patient room and were instructed to obtain a routine set of vital signs as they would during normal clinical workflow. Limb restriction information was clearly documented within the history of present illness section of the chart. The chart included documentation stating that the patient was receiving dialysis through an upper extremity arteriovenous fistula and that BP cuff placement was not permitted on the affected arm. The chart-only condition was intended to represent passive documentation of a limb restriction without the presence of an external visual reminder. No time limit was imposed for chart review or task completion. The simulated patient remained passive throughout the encounter and provided no verbal cues or reminders regarding limb restrictions. A standard rolling BP machine was positioned outside the room and brought into the simulated encounter by the participant. Participants were observed silently without intervention during each scenario. The simulated environment was standardized across participants, including patient positioning, equipment availability, and room setup. Representative images of the simulation setup are shown in Figure 2.

A wrong-limb BP attempt was defined as any initial placement or attempted placement of the BP cuff on the restricted limb. An attempt was recorded when the participant first applied or initiated application of the BP cuff to the restricted extremity before any self-correction occurred. Completion of BP measurement or cuff inflation was not required for an event to be recorded. If a participant recognized and corrected the action before cuff inflation, the event was still recorded as an incorrect initial attempt and as spontaneous error recognition and correction.

A single observer was present during each simulation scenario and recorded study outcomes on paper data collection forms. Video recording and assessment by multiple observers were not performed.

The primary outcome was the proportion of wrong-limb BP cuff placement attempts in each study condition. This outcome was intended to capture a near-miss safety event rather than a completed wrong-limb BP measurement. Secondary outcomes included spontaneous recognition and correction of an incorrect initial attempt prior to BP cuff inflation.

The target sample size of 50 participants was selected pragmatically before study initiation based on study feasibility and participant availability, with the expectation of a substantial reduction in wrong-limb BP cuff placement attempts associated with the limb alert sleeve intervention. Because each participant completed both study conditions, paired statistical analysis was performed. McNemar’s test was used to compare paired categorical outcomes between conditions. Statistical significance was defined as p < 0.05.

## Results

A total of 50 participants completed both study conditions. Participant demographics are summarized in Table 1. Participants represented a range of clinical roles and levels of clinical experience.

**Table 1:** Participant Demographics

Characteristic	Participants (n=50)
Clinical Role	
Nurses	40 (80%)
Medical Assistants	5 (10%)
Technicians	5 (10%)
Gender	
Female	31 (62%)
Male	19 (38%)
Years of Clinical Experience	
<5 Years	26 (52%)
5–10 Years	15 (30%)
>10 Years	9 (18%)

Wrong-limb BP cuff placement attempts occurred in 30 of 50 encounters (60%) in the chart-only condition compared with 1 of 50 encounters (2%) in the sleeve-present condition, representing a statistically significant reduction in wrong-limb cuff placement attempts with use of the limb alert sleeve (p < 0.0001) (Table 2).

**Table 2:** Summary of Wrong-Limb Blood Pressure Cuff Placement Attempts Paired absolute reduction in wrong-limb BP cuff placement attempts with the sleeve-present condition: 58.0% (95% CI: 44.3%–71.7%; p < 0.0001)

Condition	Wrong-Limb Attempts (n)	Total (n)	Error Rate (%)
				Chart-Only	30	50	60%
Sleeve-Present	1	50	2%				

Paired within-subject analysis demonstrated that 29 participants who initially attempted incorrect cuff placement in the chart-only condition did not repeat the error in the sleeve-present condition. One participant demonstrated a wrong-limb attempt in both conditions, while 20 participants demonstrated no wrong-limb attempts in either condition (Table 3). Although participant demographics were recorded descriptively, subgroup-specific outcome data were not collected prospectively, and formal subgroup analyses could not be performed.

**Table 3:** Paired Within-Subject Comparison of Wrong-Limb BP Cuff Placement Attempts Between Study Conditions

	Sleeve-Present Wrong-Limb Attempt	Sleeve-Present No Wrong-Limb Attempt
Chart-Only Wrong-Limb Attempt	1	29
Chart-Only No Wrong-Limb Attempt	0	20

The paired absolute reduction in wrong-limb BP attempts associated with the sleeve-present condition was 58.0% (95% CI: 44.3%-71.7%), with an exact McNemar’s test demonstrating statistical significance (p < 0.0001).

The single wrong-limb attempt observed in the sleeve-present condition was recognized and corrected prior to cuff inflation.

## Discussion

In this simulated clinical environment, use of a high-visibility limb alert sleeve significantly reduced wrong-limb BP cuff placement attempts compared with chart-based documentation alone. The observed reduction from 60% to 2% highlights an important limitation of passive chart-based safety measures and supports the effectiveness of immediate visual cues at the point of care.

These findings are consistent with broader human factors principles suggesting that passive documentation systems may be less reliable than environment-based visual cues in preventing procedural errors [6,7]. Prior studies evaluating visual reminder systems in healthcare have similarly demonstrated improved adherence to safety protocols and reductions in preventable errors through the use of immediate environmental cues [8-10]. Workflow interruptions and competing clinical demands have also been shown to increase the likelihood of procedural oversight and safety-related errors in healthcare settings [11]. In fast-paced clinical environments, clinicians may bypass chart review or overlook documented restrictions, whereas a visible physical marker positioned directly on the affected limb serves as an immediate reminder during task performance.

The single incorrect initial attempt observed in the sleeve-present condition was recognized and corrected before cuff inflation, suggesting that the sleeve may also facilitate rapid error recognition when an incorrect action is initiated. However, the study was primarily designed to evaluate the prevention of wrong-limb BP attempts rather than corrective behavior specifically.

This intervention is notable for its straightforward design and visibility at the point of care. Electronic alerts may contribute to alert fatigue in some settings, whereas a physical sleeve provides a continuous visual reminder without requiring additional workflow steps [6]. Similar workflow-based safety interventions have been shown to improve clinician adherence while minimizing disruption to routine patient care activities [5,7,10].

Several limitations should be considered. First, the study was conducted in a simulated clinical environment, which may not fully replicate the complexity, competing demands, and workflow interruptions encountered in routine patient care. Although simulation-based patient safety studies remain useful for evaluating workflow behaviors, identifying latent safety threats, and assessing system-level interventions before implementation in live clinical settings [12,13], the observed reduction in wrong-limb cuff placement attempts may not directly translate to real-world practice. In addition, the relatively high rate of wrong-limb attempts observed in the chart-only condition may have been influenced by characteristics of the simulation environment, including reliance on passive chart documentation and the absence of other clinical reminders that may be present during routine patient care.

Second, participant behavior may have been influenced by awareness of observation, consistent with the Hawthorne effect [14]. Furthermore, outcomes were assessed by a single unblinded observer, which may have introduced observer bias.

Third, although the within-subject crossover design reduced interparticipant variability, carryover effects remain possible. Participants were not informed of the study hypothesis, and no feedback was provided between conditions; however, exposure to the first scenario may have influenced performance during the second scenario.

Finally, although participant demographics were recorded descriptively, subgroup-specific outcome data were not collected prospectively, and the study was not powered for formal subgroup analyses based on clinical role, years of experience, or other participant characteristics.

Future studies involving larger and more diverse clinical populations are warranted to evaluate the generalizability of these findings and to assess the effectiveness of limb alert sleeves in real-world healthcare settings. Future implementation studies should evaluate integration with existing clinical workflows, including coordination with electronic medical record documentation, nursing handoff processes, infection control considerations, patient acceptance, and responsibility for sleeve placement and removal.

## Conclusions

In this simulated clinical study, high-visibility limb alert sleeves significantly reduced wrong-limb BP cuff placement attempts compared with chart-based documentation alone. By providing an immediate visual cue at the point of care, the intervention may help address an important gap in recognition of limb restrictions during routine clinical workflow.

Given its straightforward design and visibility at the point of care, the limb alert sleeve may represent a promising strategy for improving recognition of limb restrictions and reducing wrong-limb BP cuff placement attempts. Further studies in real-world clinical settings are warranted to confirm these findings and evaluate long-term effectiveness across broader patient populations.
